# Supplementary material for: Sonification of network traffic flow for monitoring and situational awareness
Source: PLoS One. 2018 Apr 19;13(4):e0195948. doi: 10.1371/journal.pone.0195948 (PMC5908141; doi:10.1371/journal.pone.0195948)
Supplement: S1 Appendix — This file shows the contents of the feature information arrays for Traffic flows and IP flows. (PDF) [file pone.0195948.s001.pdf]

S1 Appendix — SoNSTAR: flow and IP flow feature information array contents

When a TCP packet is received, the Feature Extractor unpacks the packet and extracts the flag status. The system has a counter which counts the packets arriving in each time window. For every packet which arrives in the time window, the Features Extractor extracts the flag status. If this flow has not been seen before it creates a new Traffic flow or IP flow if it does not already exist; if the flow already exists then it updates the flag counts for the two types of TCP protocol flows as follows.

1 Traffic Flows

Traffic Flows identified by `src addr`, `src port`, `dst addr` and `dst port` are followed by columns with flag status counts. SoNSTAR retains the information in an array carrying all feature information extracted from the packets in each time window for each Traffic flow as per Table 1.

Table 1: Feature information array: Traffic flow

Element	Label	Description
1	**Flow Counter**	This represents the number of flows in each time window.
2	**Address 1**	This is one of the IP addresses of the flow which changes to be the source or destination according to the side sending or receiving the packet.
3	**Address 2**	This is one of the IP addresses of the flow which changes to be the source or destination according to the side sending or receiving the packet.
4	**Port 1**	This is one of the ports of the flow which changes to be the source or destination according to the side sending or receiving the packet.
5	**Port 2**	This is one of the ports of the flow which changes to be the source or destination according to the side sending or receiving the packet.
6	**FIN Out**	Counts of outgoing FINs packets, which represent the total counts of outgoing packets which hold FIN status set to 1 and the status of other flags is set to 0.
7	**FIN In**	Counts of incoming FINs packets., which represent the total counts of incoming packets which hold FIN status set to 1 and the status of other flags is set to 0.
8	**SYN Out**	Counts of outgoing SYN packets, which represent the total counts of outgoing packets which hold SYN status set to 1 and the status of other flags is set to 0.
9	**SYN In**	Counts of incoming SYN packets, which represent the total counts of incoming packets which hold SYN status set to 1 and the status of other flags is set to 0.
10	**SYN ACK Out**	Counts of outgoing SYN-ACKs packets, which represent the total counts of outgoing packets which hold SYN and ACK status set to 1 and the status of other flags is set to 0.
11	**SYN ACK In**	Counts of incoming SYN-ACKs packets, which represent the total counts of incoming packets which hold SYN and ACK status set to 1 and the status of other flags is set to 0.
12	**RST Out**	Counts of outgoing RSTs packets, which represent the total counts of outgoing packets which hold RST status set to 1 and the status of other flags is set to 0.
13	**RST In**	Counts of incoming RSTs packets, which represent the total counts of incoming packets which hold RST status set to 1 and the status of other flags is set to 0.
14	**ACK Out**	Counts of outgoing ACKs packets, which represent the total counts of outgoing packets which hold ACK status set to 1 and the status of other flags is set to 0.
15	**ACK In**	Counts of incoming ACKs packets, which represent the total counts of incoming packets which hold ACK status set to 1 and the status of other flags is set to 0.
16	**PSH Out**	Counts of outgoing PSH packets, which represent the total counts of outgoing packets which hold PSH status set to 1 and the status of other flags is set to 0.
17	**PSH In**	Counts of incoming PSH packets, which represent the total counts of incoming packets which hold PSH status set to 1 and the status of other flags is set to 0.
18	**PSH ACK Out**	Counts of outgoing PSH-ACK packets, which represent the total counts of outgoing packets which hold PSH status set to 1 and ACK status set to 1 and the status of other flags is set to 0.

Continued on next page

Table 1 – *Continued from previous page*

Element	Label	Description
19	**PSH ACK In**	Counts of incoming PSH-ACK packets, which represent the total counts of incoming packets which hold PSH status set to 1 and ACK status set to 1 and the status of other flags is set to 0.
20	**URG Out**	Counts of outgoing URG packets, which represent the total counts of outgoing packets which hold URG status set to 1 and the status of other flags is set to 0.
21	**URG In**	Counts of incoming URG packets, which represent the total counts of incoming packets which hold URG status set to 1 and the status of other flags is set to 0.

2 IP flows

IP flow identified by `src addr` and `dst addr` are followed by columns of flag status counts. SoNSTAR retains the information in an array carrying all feature information extracted from the packets in each time window for each *IP flow* as per Table 2.

Table 2: Feature information array: IP-flow

Element	Label	Description
1	**IP Flow Counter**	This represents the number of IP flows in each time window.
2	**Address 1**	This is one of the IP addresses of the flow which changes to be the source or destination according to the side sending or receiving the packet.
3	**Address 2**	This is one of the IP addresses of the flow which changes to be the source or destination according to the side sending or receiving the packet.
4	**FIN Out IPs**	Counts of outgoing FINs packets between two IP addresses for whole ports, which represent the total counts of outgoing packets which hold FIN status set to 1 and the status of other flags is set to 0.
5	**FIN In IPs**	Counts of incoming FINs packets between two IP addresses for whole ports, which represent the total counts of incoming packets holds FIN status set to 1 and the status of other flags is set to 0.
6	**SYN Out IPs**	Counts of outgoing SYN packets between two IP addresses for whole ports, which represent the total counts of outgoing packets which hold SYN status set to 1 and the status of other flags is set to 0.
7	**SYN In IPs**	Counts of incoming SYN packets between two IP addresses for whole ports, which represent the total counts of incoming packets which hold SYN status set to 1 and the status of other flags is set to 0.
8	**SYN ACK Out IPs**	Counts of outgoing SYN-ACKs packets between two IP addresses for whole ports, which represent the total counts of outgoing packets which hold SYN and ACK status set to 1 and the status of other flags is set to 0.
9	**SYN ACK In IPs**	Counts of incoming SYN-ACKs packets between two IP addresses for whole ports, which represent the total counts of incoming packets which hold SYN and ACK status set to 1 and the status of other flags is set to 0.
10	**RST Out IPs**	Counts of outgoing RSTs packets between two IP addresses for whole ports, which represent the total counts of outgoing packets which hold RST status set to 1 and the status of other flags is set to 0.
11	**RST In IPs**	Counts of incoming RSTs packets between two IP addresses for whole ports, which represent the total counts of incoming packets which hold RST status set to 1 and the status of other flags is set to 0.
12	**ACK Out IPs**	Counts of outgoing ACKs packets between two IP addresses for whole ports, which represent the total counts of outgoing packets which hold ACK status set to 1 and the status of other flags is set to 0.
13	**ACK In IPs**	Counts of incoming ACKs packets between two IP addresses for whole ports, which represent the total counts of incoming packets which hold ACK status set to 1 and the status of other flags is set to 0.
14	**PSH Out IPs**	Counts of outgoing PSH packets between two IP addresses for whole ports, which represent the total counts of outgoing packets which hold PSH status set to 1 and the status of other flags is set to 0.
15	**PSH In IPs**	Counts of incoming PSH packets between two IP addresses for whole ports, which represent the total counts of incoming packets which hold PSH status set to 1 and the status of other flags is set to 0.
16	**PSH ACK Out IPs**	Counts of outgoing PSH-ACK packets between two IP addresses for whole ports, which represent the total counts of outgoing packets which hold PSH status set to 1 and ACK status set to 1 and the status of other flags is set to 0.

Continued on next page

Table 2 – *Continued from previous page*

Element	Label	Description
17	**PSH ACK In IPs**	Counts of incoming PSH-ACK packets between two IP addresses for whole ports, which represent the total counts of incoming packets which hold PSH status set to 1 and ACK status set to 1 and the status of other flags is set to 0.
18	**URG Out IPs**	Counts of outgoing URG packets between two IP addresses for whole ports, which represent the total counts of outgoing packets which hold URG status set to 1 and the status of other flags is set to 0.
19	**URG In IPs**	Counts of incoming URG packets between two IP addresses for whole ports, which represent the total counts of incoming packets which hold URG status set to 1 and the status of other flags is set to 0.
20	**URG PSH FIN Out IPs**	Counts of outgoing URG-PSH-FIN packets between two IP addresses for all ports, which represent the total counts of outgoing packets which hold URG and PSH and FIN status set to 1 and the status of other flags is set to 0.
21	**URG PSH FIN In IPs**	Counts of incoming URG-PSH-FIN packets between two IP addresses for wholeall ports, which represent the total counts of incoming packets which hold URG and PSH and FIN status set to 1 and the status of other flags is set to 0.
22	**NULL Out IPs**	Counts of outgoing NULL packets between two IP addresses for all ports, which represent the total counts of outgoing packets which hold all flags with status set to 0.
23	**NULL In IPs**	Counts of incoming NULL packets between two IP addresses for all ports, which represent the total counts of incoming packets which hold all flags with status set to 0.
24	**LAND Out IPs**	Counts of outgoing LAND packets between two IP addresses for all ports, which represent the total counts of outgoing packets which have the same source and destination IP addresses.
25	**LAND In IPs**	Counts of incoming LAND packets between two IP addresses for all ports, which represent the total counts of incoming packets which have the same source and destination IP addresses.
