# Supplementary material for: Sonification of network traffic flow for monitoring and situational awareness
Source: PLoS One. 2018 Apr 19;13(4):e0195948. doi: 10.1371/journal.pone.0195948 (PMC5908141; doi:10.1371/journal.pone.0195948)
Supplement: S2 Appendix — This file shows the contents of the questionnaire used in this experiment for evaluation. (PDF) [file pone.0195948.s002.pdf]

Sonification vs. Visualisation Questionnaire

Welcome to this very important survey with which we researchers want to learn from your experience of using visualisation only (Snort) and sonification (SoNSTAR) tools for network monitoring purpose to support situational awareness. Thank you for filling it all out.
Please note that the experiment contain three sections one for each task condition and you should fill the right section for each task.

About you

1. **Your name**: ______________________
2. **Your Gender**: ○ Male ○ Female
3. **How old are you?** I am ________ years old.
4. **What is your level of education?** I am ________ student.
5. **What is your specialty?** I am ______________
6. **What is your studying department?** I am in the ________________ .
7. **What is your year of study?** ○ first ○ second ○ third ○ forth
8. **Are you in a good mood right now to take this expermiment?** ○ absolutely ○ not really

Monitoring and Detection Tasks:

9. **Please for each task condition check the boxes of Connection testing and attacks**.

Sl.	Task Condition	A- Snort	B- SoNSTAR	C- Snort and SoNSTAR
9.0	**Connection Testing**			
1.1	ICMP ping	Yes No ☐ ☐	Yes No ☐ ☐	Yes No ☐ ☐
9.0	**Port Scan**			
1.2	SYN Related port scan	Yes No ☐ ☐	Yes No ☐ ☐	Yes No ☐ ☐
1.3	FIN port scan	Yes No ☐ ☐	Yes No ☐ ☐	Yes No ☐ ☐
1.4	XMAS port scan	Yes No ☐ ☐	Yes No ☐ ☐	Yes No ☐ ☐
1.5	NULL port scan	Yes No ☐ ☐	Yes No ☐ ☐	Yes No ☐ ☐
9.0	**DoS and DDoS**			
1.6	SYN flood	Yes No ☐ ☐	Yes No ☐ ☐	Yes No ☐ ☐
1.7	DDoS or DoS spoofed IPs	Yes No ☐ ☐	Yes No ☐ ☐	Yes No ☐ ☐

Monitoring Evaluation Tasks:

Please evaluate Snort (Visual Only)

10a. Mental Demand Rate 1 ○—○—○—○—○—○—○—○—○—○ 10

10b. Temporal Demand Rate 1 ○—○—○—○—○—○—○—○—○—○ 10

10c. Physical Demand Rate 1 ○—○—○—○—○—○—○—○—○—○ 10

10d. Performance Rate 1 ○—○—○—○—○—○—○—○—○—○ 10

10e. Effort Rate 1 ○—○—○—○—○—○—○—○—○—○ 10

10f. Frustration Rate 1 ○—○—○—○—○—○—○—○—○—○ 10

10g. Detection Confidence Rate 1 ○—○—○—○—○—○—○—○—○—○ 10

10h. Ease of Use Rate 1 ○—○—○—○—○—○—○—○—○—○ 10

10i. Visual Fatigue Rate 1 ○—○—○—○—○—○—○—○—○—○ 10

Please evaluate SoNSTAR (Sonifiction)

**11a.**	**Mental Demand Rate**	1 ○—○—○—○—○—○—○—○—○—○ 10
**11b.**	**Temporal Demand Rate**	1 ○—○—○—○—○—○—○—○—○—○ 10
**11c.**	**Physical Demand Rate**	1 ○—○—○—○—○—○—○—○—○—○ 10
**11d.**	**Performance Rate**	1 ○—○—○—○—○—○—○—○—○—○ 10
**11e.**	**Effort Rate**	1 ○—○—○—○—○—○—○—○—○—○ 10
**11f.**	**Frustration Rate**	1 ○—○—○—○—○—○—○—○—○—○ 10
**11g.**	**Detection Confidence Rate**	1 ○—○—○—○—○—○—○—○—○—○ 10
**11h.**	**Ease of Use Rate**	1 ○—○—○—○—○—○—○—○—○—○ 10
**11i.**	**Sound Fatigue Rate**	1 ○—○—○—○—○—○—○—○—○—○ 10

Please evaluate best task condition

12. What is best for you to use for detection? ○ Snort ○ SoNSTAR ○ Both Together

Please evaluate the following tools

13a. Snort horrible ○—○—○—○—○ fantastic

13b. SoNSTAR horrible ○—○—○—○—○ fantastic

About this Experiment

14. Do you like this experiment? ○ Yes ○ No

15. Is it really worth your future participation? ○ Guess so. ○ Probably not. ○ Don't know.

16a. Please describe your first impression.

____

____

16b. In case you would like some more lines to write, here they are:

____

____

____

____

Thank you for your feedback and participation
