# Supplementary material for: Sonification of network traffic flow for monitoring and situational awareness
Source: PLoS One. 2018 Apr 19;13(4):e0195948. doi: 10.1371/journal.pone.0195948 (PMC5908141; doi:10.1371/journal.pone.0195948)
Supplement: S5 Appendix — This file shows the contents of the consent form used in this experiment. (PDF) [file pone.0195948.s005.pdf]

Faculty of Engineering and Environment

RESEARCH PARTICIPANT CONSENT FORM

**Name of participant**	
**Researcher's name**	
**Programme of study**	
**Supervisor's name**	

Brief description of nature of research and involvement of participant: The research is about increasing the situational awareness of real time network monitoring tools and because situational awareness of network activity needs to be maintained to ensure an appropriate response to attacks and the efficient management of network resources. We researchers want to learn from your experience of using visualisation only (Snort) and sonification (SoNSTAR) tools for network monitoring purpose to support situational awareness. AS participant, you will be asked to perform three tasks of network monitoring using both tools and will be asked to fill out questionnaire designed to collect specific information for each task condition.

****Statement of participant consent (please tick as appropriate)**

I confirm that:

I have been briefed about this research project and its purpose and agree to participate ☐

I have been given the opportunity to ask questions about the project and my participation. ☐

I voluntarily agree to participate in the project. ☐

I understand I can withdraw at any time without giving reasons and that I will not be penalised for withdrawing nor will I be questioned on why I have withdrawn ☐

I have discussed any requirement for anonymity or confidentiality with the researcher ☐

I agree to being audio recorded/filmed/photographed ☐

****Specific requirements for anonymity, confidentiality, data storage, retention and destruction**

Participant:
Signed: …………………………………………………… Date: ……………………………….

Researcher:
Signed: …………………………………………………… Date: ………………………………..
